# The Creation and Physiological Relevance of Divergent Hydroxylation Patterns in the Flavonoid Pathway

**DOI:** 10.3390/ijms11020595

**Published:** 2010-02-04

**Authors:** Heidi Halbwirth

**Affiliations:** Institut für Verfahrenstechnik, Umwelttechnik und Technische Biowissenschaften, Technische Universität Wien, Getreidemarkt 9/1665, A-1060 Wien, Austria; E-Mail: hhalb@mail.zserv.tuwien.ac.at; Tel.: +43-1-58801-17311; Fax: +43-1-58801-17399

**Keywords:** plant, flavonoid, chalcone, hydroxylation, cytochrome P450 dependent monooxygenase, 2-oxoglutarate-Fe(II)-dependent dioxygenase, oxidoreductase

## Abstract

Flavonoids and biochemically-related chalcones are important secondary metabolites, which are ubiquitously present in plants and therefore also in human food. They fulfill a broad range of physiological functions *in planta* and there are numerous reports about their physiological relevance for humans. Flavonoids have in common a basic C_6_-C_3_-C_6_ skeleton structure consisting of two aromatic rings (A and B) and a heterocyclic ring (C) containing one oxygen atom, whereas chalcones, as the intermediates in the formation of flavonoids, have not yet established the heterocyclic C-ring. Flavonoids are grouped into eight different classes, according to the oxidative status of the C-ring. The large number of divergent chalcones and flavonoid structures is from the extensive modification of the basic molecules. The hydroxylation pattern influences physiological properties such as light absorption and antioxidative activity, which is the base for many beneficial health effects of flavonoids. In some cases antiinfective properties are also effected.

## 1. Introduction

Flavonoids were the first secondary metabolites to be studied, with some flavonoid classes, starting with the visibly detectable anthocyanins, having been known for a long time [1]. The distribution in the plant kingdom and the presence of flavonoids in different plant tissues has been widely studied [2]. In the seventies and eighties of the past century, most steps in the biosynthetic pathway that lead to the main flavonoid classes were successfully elucidated [3]. In the nineties there was an explosion in the amount of literature focusing on different aspects of flavonoids, including genetics, horticulture, plant biochemistry, biotechnology, nutrition, pharmacognosy and phytomedicine. The aim of this article is to summarize the knowledge about the introduction of hydroxyl groups in the flavonoid pathway and to review possible structure-activity relationships clearly dependant on the hydroxylation pattern of flavonoids.

## 2. Flavonoids: Structures and Physiological Functions

**Figure 1.**
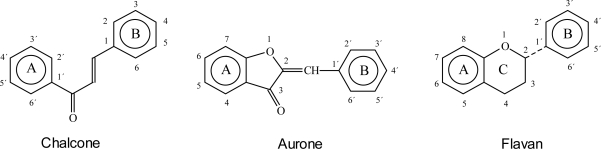
The basic structures of chalcone, aurone and flavan.

Flavonoids have in common a basic C_6_-C_3_-C_6_ skeleton structure consisting of two aromatic rings (A and B) and a heterocyclic ring (C) containing one oxygen atom. This structure is known as flavan (Figure 1, right). Formerly, chalcones and aurones (Figure 1, left and center) were counted among the so-called minor flavonoids [4]. Nowadays they are regarded as a biochemically-related but separate group because their chemical structure cannot be derived from the typical flavan structure. Hence, ring numbering in chalcones and flavonoids is divergent and position 3 of chalcones corresponds to position 3′ of flavonoids and aurones [5] (Figure 1).

**Figure 2.**
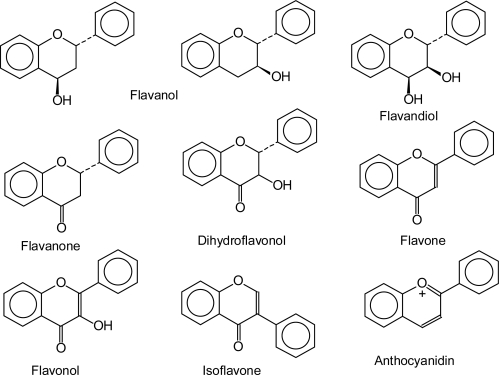
The basic structures of flavonoid classes.

Flavonoids are ubiquitously present in the plant kingdom and are even found in some fungal species [6–8], but not all flavonoid classes are found in all plant groups [9]. In 1999, the most comprehensive register of flavonoids listed 6,467 naturally occurring compounds [2] and the identification of novel structures from various plant sources is ongoing. Flavonoids are grouped into eight different flavonoid classes, defined according to the oxidative status and the number and type of substituents on the heterocyclic ring (Figure 2).

The abundance of natural flavonoid structures results from the extensive modification of the basic flavonoid classes. Thus, additional hydroxyl groups are usually present in rings A and B. Hydroxyl groups can be further methylated or glycosylated and the flavonoid glycosides frequently carry acyl groups at their sugar moieties. In some cases the sugar moiety can be linked to the flavonoid structure via a C-C bond [2].

In the early 1960s, flavonoids were widely regarded as metabolic waste products stored in the plant vacuoles [10]. Since then, knowledge about the numerous contributions that flavonoids make to the fitness and survival of plants has continuously increased. It is now accepted that flavonoids can multitask with respect to their physiological functions in plants. They protect against abiotic and biotic stress [11,12], act as attracting signals for soil bacteria [13] and seed and pollen dispersers [14] and as deterring signals for herbivores [15] and contribute to plant growth and fertility [11,16,17].

The subcellular localization of flavonoids and of the underlying pathway has been investigated in detail. Although flavonoids are believed to be formed in the cytosol, they are mainly stored in the vacuoles. This requires a so far unknown flavonoid transport mechanism from the biochemical formation site to the accumulation site. It is assumed that different transport mechanisms are used in the plant kingdom [18] and there is evidence that GST (gluthathione *S-*transferase)-like proteins and MATE (multidrug and toxic compound extrusion)-type transporters could be involved [19]. The majority of the physiological functions such as pigmentation, UV and light protection and pathogen defense are consistent with an accumulation in vacuoles, whereas others are based on the presence in the cytosol, e.g., the modulation of auxin movement [20]. Storage in the vacuoles requires the modification of basic flavonoid structures. In particular, glycosylation increases water solubility and subsequent acylation is a precondition for the uptake and trapping of flavonoids in the vacuoles [21,22]. Cytosolic flavonoids are not well investigated. Because flavonoids are reactive and potentially cytotoxic compounds, free diffusion in the cytoplasm seems unlikely. Recently, the presence of flavanols was demonstrated in the nucleus of different plants which could be associated to DNA protection [23,24].

Flavonoids are wide-spread in plants and therefore also in human foods and drinks. The daily intake of flavonoids however, depends on the composition and is estimated to range from 23 mg up to more than 1 g. Food and drinks particularly high in flavonoid concentrations are onions, fruits, green tea and cocoa [25–27]. Flavonoids have been suggested to play a manifold role in disease prevention, such as reducing the risks of cancer [28], cardiovascular disease [29,30], diabetes [31], brain diseases [32], cataracts [33] and other functional declines associated with ageing [34]. Many of the observed preventative effects are based on the radical scavenging and antioxidant properties of flavonoids and on their interaction with various enzymes [29,35]. However, their disposition in humans and their range of concentration present in human plasma—either as aglycones or as glycosylated metabolites—is still a matter of debate [27,36–39]. For a more detailed summary of the potential effects of flavonoids in plants and in human nutrition refer to [40].

## 3. Overview of the Flavonoid Pathway

**Figure 3.**
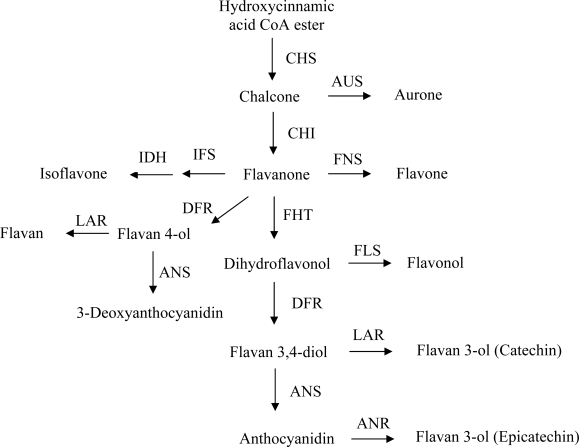
The flavonoid pathway. Abbrev: ANR: anthocyanidine reductase, ANS: anthocyanidine synthase, AUS: aurone synthase, CHI: chalcone isomerase, CHS: chalcone synthase, DFR: dihydroflavonol 4-reductase, FHT: flavanone 3-hydroxylase, FLS: flavonol synthase, FNS: flavone synthase, IDH: 2-hydroxyisoflavanone dehydratase IFS: 2-hydroxyisoflavanone synthase, LAR: leucoanthocyanidin reductase.

Although the term ‘flavonoid pathway’ suggests a linear biochemical connection from some starting point to an end product via a certain number of intermediates, flavonoid biosynthesis represents a complex grid rather than a simple chain. An overview on the flavonoid biosynthesis is given in Figure 3 [3].

The consecutive co-operation of five different enzymes–chalcone synthase (CHS), chalcone isomerise (CHI), flavanone 3-hydroxylase (FHT, synonym: F3H), dihydroflavonol 4-reductase (DFR), and anthocyanidin synthase (ANS) – finally leads to the formation of anthocyanidins. However, all occurring intermediates are also precursors for the formation of other flavonoid classes. The key reaction is catalyzed by the enzyme CHS, which was first demonstrated in cell cultures of *Petroselinum crispum* [41]. The preflavonoid precursors which are condensed by CHS are derived from the carbohydrate metabolism. Stereospecific cyclization of the chalcone molecule leads to the formation of flavanone, the first flavonoid, which is the immediate precursor of flavones, isoflavones, flavan 4-ols and dihydroflavonols. The reaction is catalyzed by CHI, an enzyme which does not require any cofactor. Flavone is derived from flavanone by the introduction of a double bond between C-2 and C-3. Two types of enzymes were described to be responsible: a 2-oxoglutarate-dependent dioxygenase, flavone synthase I (FNS I) and a cytochrome P450-dependent monooxygenase, flavone synthase II (FNS II). Co-action of 2-hydroxyisoflavanone synthase (IFS) and a dehydratase (IDH) provides isoflavone from flavanone. Alternatively, hydroxylation in position 3 of flavanone, which is catalyzed by the 2-oxoglutarate-dependent dioxygenase FHT, provides dihydroflavonol, which is the direct intermediate for flavonol and flavan-3,4-diol (leucoanthocyanidin) formation. The introduction of a double bond between C-2 and C-3 of dihydroflavonol generates flavonol. This step is catalysed by flavonol synthase (FLS), a 2-oxoglutarate-dependent dioxygenase. Alternatively, dihydroflavonol is converted to flavan-3,4-diol by DFR, a key enzyme at a branching point of the flavonoid pathway. It reduces dihydroflavonols to leucoanthocyanidins (flavan-3,4-diols), which are the direct precursors for the formation of both catechins and anthocyanidins (Figure 3). Additionally, DFRs also possess flavanone 4-reductase (FNR) activity and can reduce flavanones to the corresponding flavan 4-ols. The leucoanthocyanidins produced by DFR can either be converted by leucoanthocyanidin recutase (LAR) to catechins, or can be the substrate for anthocyanidine synthase (ANS) to produce anthocyanidins. The latter can either be stabilized by glycosylation to form anthocyanins or, alternatively, can be reduced by anthocyanidine reductase ANR to form epicatechins. LAR shows a broad substrate specificity and can further reduce flavan-4-ols to flavans [42]. The formation of proanthocyanidins is still a puzzle [43].

The flavonoid pathway is based on a large number of soluble enzymes present in the cytosol including ANR, ANS, CHI, CHS, DFR, FHT, FLS, LAR and modifying enzymes such as glucosyl transferases and methyl transferases. In addition, many hydroxylating enzymes are anchored in the membranes of the endoplasmic reticulum (ER). Depending on the plant species, FNS can be present as soluble or membrane-bound enzymes. It is assumed that flavonoid biosynthesis is organized in multienzyme complexes [44] which are loosely associated with the cytoplasmic face of the ER membrane [45,46]. Recently, it was also suggested that at least one biosynthetic step of the flavonoid pathway - the formation of aurones - is performed in the vacuoles [47] and CHS and CHI were shown to be present in the nucleus of several cell types in *Arabidopsis thaliana* [48]. Therefore, the *in situ* formation of nucleus-located flavonoids might be possible but has not been demonstrated so far. This provides the first evidence that flavonoid formation could be localized in different parts of the cell [19].

## 4. The Creation of the Hydroxylation Patterns of Flavonoids

**Figure 4.**
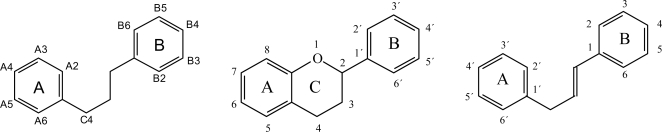
Simplified C_6_-C_3_-C_6_ structure (left) of flavonoids (centre) and chalcones (right).

The following generalization can be made regarding the hydroxylation pattern of the C_6_-C_3_-C_6_ structure (Figure 4), which is common to flavonoids and chalcones (modified from [49]):

1. The kind and position of hydroxylation in the naturally occurring C_6_-C_3_ compounds is similar to that found in the C_6_(B)-C_3_-fragment of the naturally-occurring flavonoids, and is usually different from that of the C_6_(A)-C_3_-fragments;
2. Hydroxylation (or a C-O-bond respectively) is always found at A-2, and in the great majority of cases, at A-2,4,6, respectively. With a few exceptions, hydroxylation always occurs at least at both A-2 and A-4;
3. Hydroxylation is known to occur at all possible positions in A; flavonoids are known which are A-2,3,4,5,6-hydroxylated;
4. The greatest number of compounds are hydroxylated at B-4 with B-3,4 and B-3,4,5 also common;
5. Hydroxylation at B-2 is known, but there is no example of B-2,6 hydroxylation;
6. The state of oxidation of the C-chain can vary; and
7. The C-4 carbon atom is, in most cases, present as a carbonyl group.

In the flavonoid pathway, hydroxyl groups in the A-, B- and C-rings are introduced at various biosynthetic stages. There is a wide range of enzymes that may catalyze hydroxylation reactions, e.g., different types of dioxygenases and monooxygenases, hydroxyl radical generating systems and enzymes showing hydroxylating side activities such as tyrosinase or certain peroxidases [50]. In the flavonoid pathway, the majority of the hydroxylating enzymes are 2-oxoglutarate-Fe(II)-ascorbate-dependent dioxygenases or cytochrome P450-dependent monooxygenases [3]. However, particular hydroxyl groups can be also obtained by reducing oxo groups or by the action of a bifunctional polyphenol oxidase [51]. Cytochrome P450-dependent monooxygenases form one of the largest protein families in higher plants and they may catalyze a large number of different reactions [52]. Both cytochrome P450-dependent monooxygenases and 2-oxoglutarate-Fe(II)-ascorbate-dependent dioxygenases use O_2_ as oxygen donator and introduce one of the oxygen atoms into the hydroxyl group of the flavonoids during the catalytic cycle. The second oxygen atom is transferred to 2-oxoglutarate in the case of 2-oxoglutarate-Fe(II)-ascorbate-dependent dioxygenases or reduced to water with NADPH as hydrogen donor in the case of cytochrome P450-dependent monooxygenases [50].

### 4.1. Introduction of Hydroxyl Groups in the Flavonoid A-ring

**Figure 5.**
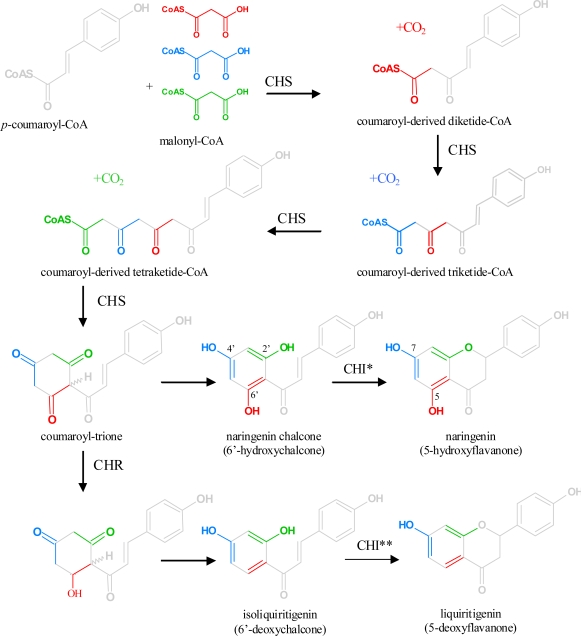
A-ring formation by CHS and creation of different hydroxylation pattern in ring A (modified from [56]). * all known CHIs catalyze this reaction, ** only specific CHIs catalyze this reaction.

There are few flavonoid structures which do not show any hydroxyl group in the A-ring or a single hydroxyl group in position 6 [2]. Such rare structures seem to be formed particularly in the Primulaceae, Rutaceae and Thymelaeaceae families. The mechanism(s) of their biochemical formation, however, is still unknown. The vast majority of flavonoids possess a basic 5,7-hydroxylation pattern of the A-ring, which is built from malonyl-CoA during the formation of the chalcone (Figure 5).

Chalcones are the first C_15_-structures of the flavonoid pathway (Figure 5). During the catalytic process, the A-ring of chalcones is assembled from 3 × 2 carbons from the three molecules of malonyl-CoA, whereas the three other carbons are removed by decarboxylation [41,53–55]. Chalcones are converted by CHI to *(2S)-*flavanones, which are the intermediates for all further flavonoid classes. CHIs of most plants show a distinct substrate specificity and accept only the common 6′-hydroxychalcones as substrates [3].

In some plants, particularly in members of the Leguminosae family, flavonoids lacking a 5-hydroxyl group are found [4]. Their formation occurs by co-action of CHS and CHR (synonyms: polyketide reductase PKR, chalcone ketide reductase). CHR catalyzes the reduction of the keto group of the coumaroyl-trione intermediate in position 6′ and after the loss of water the 6′-deoxychalcones are obtained [56], which are the precursors for the formation of all 5-deoxyflavonoid classes. The ability of plants to form 5-deoxyflavonoids primarily depends on the presence of CHR and a specific CHI, which accepts 6′-deoxychalcones as substrates beside the common 6′-hydroxychalcones. All further downstream enzymes from the flavonoid pathway seem to accept 5-deoxyflavonoids apart from their natural 5-hydroxyflavonoid substrates. Conversion rates, however, depend on the enzyme type and origin [57–59]. Because of the lacking hydroxyl group in position 5, 5-deoxyflavan 3,4-diols are much more stable than their corresponding 5-hydroxycounterparts.

**Figure 6.**
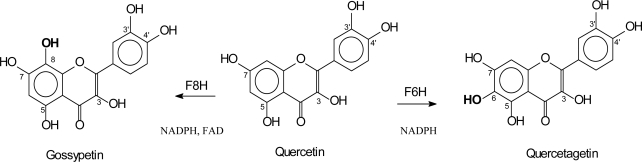
Introduction of additional hydroxyl groups in the A-ring of flavonoids.

Additional hydroxyl groups in ring A can be introduced at positions 6 and 8 (Figure 6). A 2-oxoglutarate-dependent dioxygenase (F6H) is responsible for the 6-hydroxylation of partially methylated flavonols in the semiaquatic weed plant *Chrysosplenium americanum* [60,61]. In contrast, F6Hs from *Tagetes patula* and *Rudbeckia hirta* require the aglycon quercetin as a substrate and were identified as a cytochrome P450-dependent monooxygenases [62,63]. Generally, the occurrence of two different enzyme systems catalyzing one and the same reaction has rarely been reported and is therefore of great biochemical interest. A cytochrome P450-dependent F6H from elicitor-treated soybean seems to be specifically involved in the biosynthesis of 6-hydroxyisoflavones. Flavanones were the preferred substrates, whereas the flavonol kaempferol was barely accepted [64].

An enzyme introducing a hydroxyl group in position 8 of flavonols and flavones was demonstrated with enzyme preparations from petals of *Chrysanthemum segetum* [65]. Flavanones, dihydroflavonols and glucosylated flavonoids were not accepted as substrates. The enzyme is localized in the microsomal fraction and requires NADPH and FAD as cofactors. F8H represents a novel type of hydroxylating enzyme in the flavonoid pathway as there seems to be no involvement of cytochrome P450 in the reaction [65].

### 4.2. Introduction of Hydroxyl Groups in the Flavonoid B-ring

The hydroxylation pattern of the B-ring is firstly determined by the C_6_-C_3_ precursor used by CHS. As a rule, *p-*coumaroyl-CoA (4-hydroxycinnamoyl-CoA) is the physiological standard precursor. The resulting basic C_15_ chalcone intermediate, naringenin chalcone, carries a hydroxyl group in position 4 and all derived flavonoid structures have the hydroxyl group in position 4′, which is found in common flavonoid structures (Figure 5). Thus, the introduction of the hydroxyl group in position 4′ is a pre-flavonoid step, which is performed by cinnamate 4-hydroxylase, a cytochrome P450-dependent monooxygenase, which catalyzes the formation of *p*-coumaric acid [66,67].

**Figure 7.**
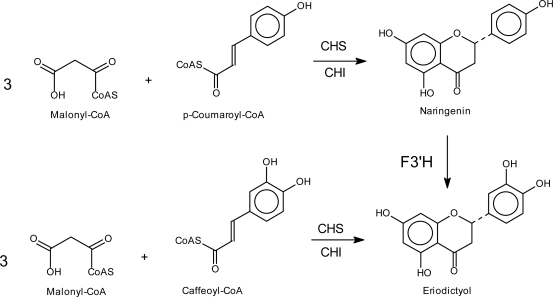
Two different possibilities to introduce the hydroxyl group in position 3′of flavonoids.

The hydroxyl group in position 3′ can be generated in two different ways (Figure 7). First, the incorporation of caffeoyl-CoA (CoA-ester of the 3,4-dihydroxycinnamic acid) instead of *p*-coumaroyl-CoA (CoA-ester of the 4-hydroxycinnamic acid) in the CHS step results in the formation of 3,4-hydroxylated chalcones which are intermediates for the corresponding 3′,4′-hydroxylated flavonoids. In most plants, CHS accepts caffeoyl-CoA as a substrate to a sufficient extent *in vitro*. However, only in a few cases caffeoyl-CoA was shown to be physiologically relevant as precursor for chalcone formation [3, 68], e.g for scarlet *Verbena hybrida* lines accumulating 10% cyanidin in the petals despite the absence of F3′H activity [69]. Second, as a rule, the additional hydroxyl group in position 3′ of ring B is introduced by specific enzymes, which can act at different levels of the pathway.

**Figure 8.**
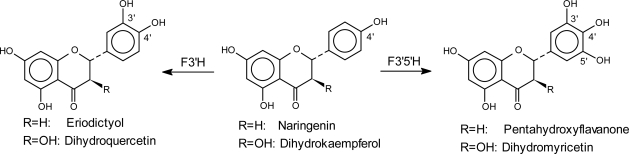
Examples of the introduction of hydroxyl groups in positions 3′ and 3′,5′ of flavonoids.

The cytochrome P450-dependent monooxygenase flavonoid 3′-hydroxylase (F3′H) (Figure 8) was first demonstrated in microsomal fractions of cell suspension cultures from *Haplopappus gracilis* [70] and was then investigated in many different plant species [3]. The enzyme was shown to accept flavanones, flavones, flavonols and dihydroflavonols as substrates [71]. Anthocyanidins, however, cannot be hydroxylated by F3′H [3]. The first *f3′h* cDNA clone was isolated from *Petunia hybrida* and functionally expressed in yeast [72].

Many plants (e.g., Asteraceae species) show a corresponding B-ring hydroxylation pattern of chalcones and flavonoids [4]. An enzyme was demonstrated in *D. variabilis* petals that catalyzes the hydroxylation of chalcones in position 3 [73], but it remained unclear whether chalcone 3-hydroxylation is performed by the common F3′H or an unknown independent enzyme. The involvement of F3′H seemed to be likely, because (i) the same position in the B-ring of flavonoids and chalcones is affected, (ii) F3′H generally shows broad substrate acceptance and (iii) the hydroxylation of both flavonoids and chalcones is catalyzed by NADPH-dependent enzymes localized in the microsomal fraction. However, common F3′Hs do not hydroxylate chalcones at position 3 [74]. Recent research has shown that chalcone 3-hydroxylation can be catalyzed to some extent by special F3′Hs with distinct structural pre-requisites [75]. In addition, some plants possess beside the common F3′Hs additional specific enzymes, which have a better ability to hydroxylate chalcones compared to their F3′H activity [76]. Such enzymes can be regarded as chalcone 3-hydroxylases (CH3Hs).

Flavonoid 3′,5′-hydroxylase (F3′5′H) is responsible for the generation of the 3′,4′,5′ hydroxylation pattern (Figure 8). It can also act at different levels in the flavonoid pathway and catalyzes 3′,5′-hydroxylation of 4′-hydroxylated flavanones, flavonols, flavones and dihydroflavonols and 5′-hydroxylation of the corresponding 3′,4′-hydroxylated structures. F3′5′H is a cytochrome P450-dependent monooxygenase [77,78]. The presence of this enzyme is an essential pre-condition for the formation of delphinidin (anthocyanidin with three hydroxyl groups in the B-ring), which is responsible for blue and violet flower coloration attracting honey bees – the main pollinators in the temperate regions. F3′5′H was first demonstrated in microsomal preparations from *Verbena hybrida* [79] and has since been isolated from approximately 50 plants, mainly angiosperms. F3′5′H was apparently recruited from F3′H before the separation of angiosperms and gymnosperms [80] as an adaptation to the requirement of blue flower coloration to attract insect pollinators. Interestingly, Asteraceae show specific F3′5′Hs, which have evolved independently from Asteraceae specific F3′Hs.

The presence of an enzyme catalyzing the introduction of a hydroxyl group in position 2′ of flavonoids is expected for *Heywoodiella oligocephala* accumulating isoetin (2′-hydroxyluteolin) derivatives as main yellow flower pigment [81,82]. However, hydroxylation in position 2′ has not been investigated so far.

### 4.3. Introduction of Hydroxyl Groups into the C-ring of Flavonoids

**Figure 9.**
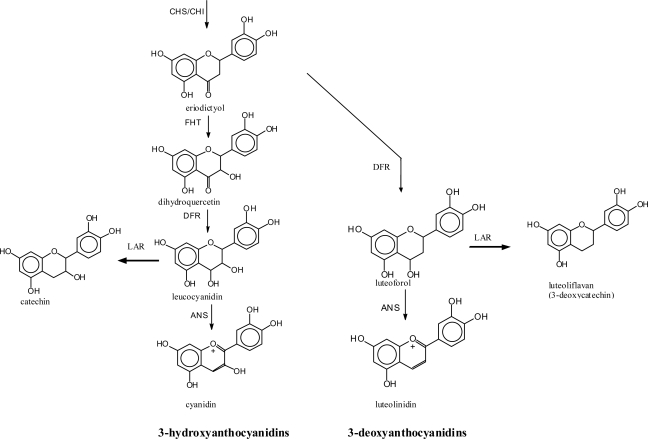
Formation of flavonoids with and without hydroxyl groups in position 3.

The majority of flavonoid classes carry a hydroxyl group in position 3 (Figure 2). The hydroxyl group is introduced at the level of flavanones by the well studied FHT, a 2-oxoglutarate, Fe(II) and ascorbate-dependent dioxygenase [83–85]. The absence of this enzyme is a precondition for the formation of the rare 3-deoxyanthocyanidins [86] (Figure 9). Absent or reduced FHT activity is observed in natural mutants [87–90] or during transient downregulation of FHT [91,92]. Similar effects can be achieved by the application of chemical compounds [57,59] or via genetic engineering [93–95]. However, the absence of FHT alone does not ensure the formation of 3-deoxyanthocyanidins in plant tissues.

The majority of flavonoid classes carry an oxo group in position 4, but in the case of flavan-3,4-diols and flavan-4-ols, a hydroxyl group is found instead. This hydroxyl group results from the reduction of the 4-oxo group of ring C. The step is catalyzed by the well studied oxidoreductase DFR [3,58,86,96–98]. As dehydrogenase, DFR catalyzes also the reverse reaction, i.e. the conversion of flavan 3,4-diols into dihydroflavonols or flavan-4-ols into flavanones in the presence of NADP^+^ [58]. The physiological relevance of the reverse DFR reaction has not been studied so far, but an impact on metabolic fluxes in the flavonoid pathway is possible.

In some plants, e.g., petunia and tobacco, DFR shows a distinct substrate specificity in terms of the hydroxylation pattern in ring B and accepts dihydroquercetin (3′,4′-hydroxylation pattern) and dihydromyricetin (3′,4′,5′-hydroxylation pattern) but not dihydrokaempferol (4′-hydroxylation pattern) as substrates [3]. As dihydroflavonols are intermediates in the formation of anthocyanins, this DFR substrate specificity determines flower color. Such plants can accumulate derivatives of cyanidin (dark red pigment; 3′,4′-hydroxylation pattern) and delphinidin (blue pigment; 3′,4′,5′-hydroxylation pattern), but scarlet flower coloration, which would be caused by the accumulation of pelargonidin derivatives (scarlet pigment; 4′-hydroxylation pattern) does not occur in these plants.

In addition, DFR also catalyses the reduction of flavanones to the corresponding flavan-4-ols (flavanone-4-reductase activity, FNR-activity), which are intermediates in the 3-deoxyanthocyanidin formation (Figure 9) [86,99]. This was first investigated with plants producing 3-deoxyanthocyanidins under natural conditions [86,89]. Physiological relevance in planta of the FNR-activity of DFR is only observed in cases where FHT activity is reduced or absent. However, all DFR enzymes tested so far catalyze the reaction [3,57,86,89,96,100–102], although dihydroflavonols are the preferred substrates. Therefore, the common 3-hydroxyflavonoids are exclusively formed as long as dihydroflavonols are available as substrates.

## 5. Structure-Activity Relationships (SARs)

The chemical features and biological activities of flavonoids are influenced by the number and nature of the substituents affiliated to the basic structures. Increasing numbers of hydroxyl groups and sugar moieties increase water solubility, whereas methylation of the hydroxyl groups decreases water solubility. Free hydroxyl groups are more reactive than glucosylated and methylated hydroxyl groups and this is essential for many of the biological activities related to the antioxidant capacity of the flavonoid structures. The biological activity frequently seems to be related to the presence of vicinal hydroxyl groups at positions 3′ and 4′, a hydroxyl group at position 3 of the C-ring, a carbonyl group at C4 of the C-ring and a C2-C3 double bond [103–110]. However, for some biological activities only one of these characteristics or other or even contrasting structural requirements are decisive [97–102]. Unfortunately few studies have systematically investigated SARs with respect to the hydroxylation pattern of flavonoids.

### 5.1. Relationship between the Structure and Light Absorbance of Flavonoids

**Figure 10.**
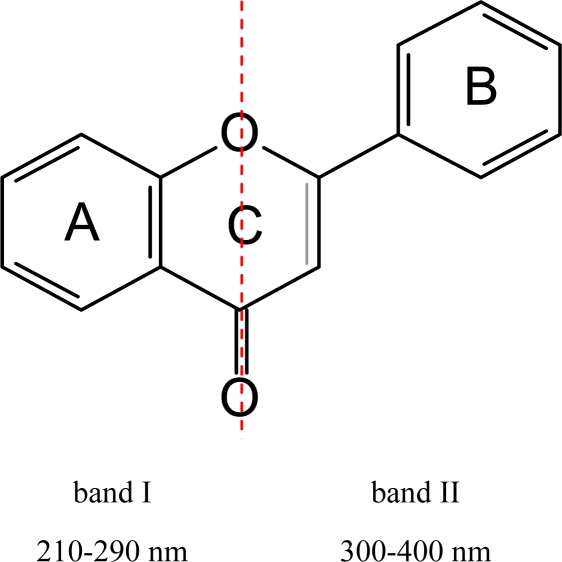
Structural base for flavonoid light absorbance.

**Figure 11.**
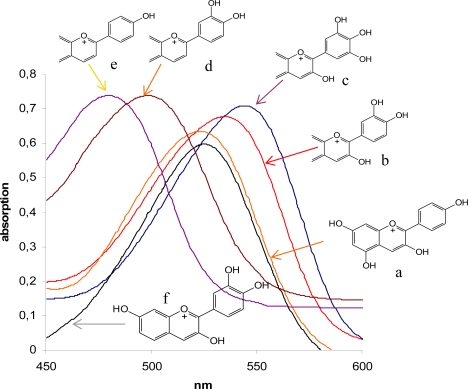
Part of the absorption spectra of anthocyanidins (a–f) with divergent hydroxylation patterns. With the exception of pelargonidin (a) and robinetinidin (f), structures of cyanidin (b), delphinidin (c), luteolinidin (d) and apigeninidin (e) are only partially shown.

**Figure 12.**
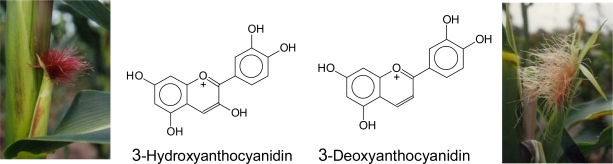
Chemical structures of cyanidin and luteolinidin and tissue colours of *Zea mays* silks accumulating the respective anthocyanidin, left: red silks accumulating common 3-hydroxyanthocyanins, right: salmon silks accumulating the 3-deoxyanthocyanins (derivatives of luteolinidin).

The UV-VIS spectrum of flavonoids typically shows a band at 210–290 nm region (band I), which is due to the absorption of the benzoyl system (A-ring), and a second band in the 300–400 nm region (band II), which is associated with the cinnamoyl system (rings B and C) [117] (Figure 10). With increasing conjugation the absorbance shifts towards higher wavelengths, therefore, chalcones, aurones and anthocyanidins have maximal absorbance in the visible part of the spectrum. In the case of anthocyanidins, band II shows its maximum around 520 nm. For many pollinating insects absorption in the UV (band I) is also relevant for colour perception because their specific colour sense makes them blind to scarlet red coloration but sensitive to the UV-range of the spectrum [118–123]. The number and type of substituents affiliated to the basic flavonoid structure has an impact on light absorbance. Additional hydroxyl groups shift the absorption maxima. The hydroxyl group in position 3 of ring C has a higher impact on the absorption maximum of band II than hydroxyl groups in rings A and B. For anthocyanidins (compare a with e and b with d in Figure 11), this results in a colour shift of 3-deoxyanthocyanidins into the yellow region (Figure 12).

**Figure 13.**
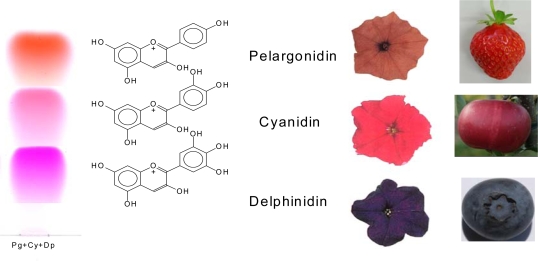
Chemical structures of the most common anthocyanidins, their spots on TLC (cellulose with H_2_O/HCl/Hac = 82/3/15 as solvent system) and the colour of plant tissues accumulating primarily the respective anthocyanidin derivatives (orange petunia is genetically modified).

With increasing numbers of hydroxyl groups at rings A and B, the absorbance shifts slightly towards higher wavelengths. Thus, pelargonidin (one hydroxyl group in the B-ring) has its maximum at 510 nm, cyanidin (two hydroxyl groups in the B-ring) at 520 nm and delphinidin (three hydroxyl groups in the B-ring) at 530 nm (Figures 11 and 13). To the human eye, this shifts the colour of the pure substances as well as of tissues in which the substances are accumulated, from bright red in the case of pelargonidin to the lilac hues of delphinidin (Figure 13). However tissue colouration by anthocyanidins may also be influenced by other factors, such as pH, co-pigmentation and metal complexation [12,14].

Cyanidin is regarded as the most primitive anthocyanidin pigment, because it is (i) the most common type found in gymnosperms, which are the anchestors of angiosperms, (ii) the most common anthocyanidins formed in leaves and stems, and (iii) the most common pigment of wind-pollinated flowers [18]. The other anthocyanidin types are regarded as being more advanced and seem to have evolved during the adaptation of angiosperms to different pollinators. The regular occurrence of bright-red pelargonidin (a in Figure 11), orange luteolinidin (d in Figure 11) and yellow apigeninidin (f in Figure 11) in tropical plants and their almost complete absence in temperate floras reflect the need for adaptation to birds as the most active pollinators in tropical habitats [14].

In contrast, flowers in temperate regions are pollinated by insects rather than by birds. This resulted in the formation of purple and blue delphinidin-based pigments (c in Figure 11) as an adaptation to the specific colour sense of insects [14]. Flavonols with additional hydroxyl groups in ring A are responsible for the formation of UV-honey guides in *Rudbeckia hirta* [14,119], but recent research has shown that the glycosylation pattern is also decisive [63]. The pigments accumulate in the inner part of the petals and this is perceived as colour pattern by pollinating insects whereas the flowers appear uniformly yellow to the human eye.

### 5.2. Relationship between the Structure and Antioxidant Activity of Flavonoids

Most of the beneficial health effects of flavonoids are ascribed to their antioxidant abilities [124]. Antioxidant activity is influenced by a mixture of properties including the capacity for reactive oxygen species (ROS) scavenging, metal ions chelation, the regeneration of other antioxidative compounds such as α-tocopherol, activation of antioxidant enzymes and the inhibition of radical-producing enzymes [124–126].

Although flavonoids are widely accepted as being potent antioxidant compounds, there is a controversy discussion about their relative contribution and SARs [126]. One of the reasons for this might be the broad spectrum of different methods used to measure antioxidant capacities and the fact that the different components influencing antioxidative behaviour cannot easily be measured separately. However, the antioxidant capacity of a compound primarily depends on the presence of dissociable protons, vicinal hydroxyl groups and conjugated double bonds [125,127,128].

**Figure 14.**
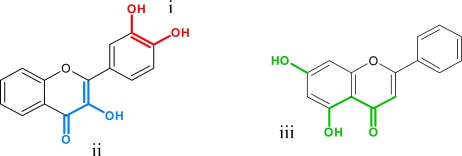
The three main structural prerequisites of flavonoids for high antioxidant potential (modified from [127]).

In the case of flavonoids, the structural indications for high antioxidant capacity seem to be (i) an *ortho* 3′, 4′-dihydroxy moiety in the B-ring (Figure 14 red), (ii) a 2,3-double bond in combination with the 4-oxo group and a hydroxyl group in position 3 (Figure 14 blue), and (iii) a *meta* 5, 7-dihydroxy moiety in the A-ring in combination with the 4-oxo group (Figure 14 green), because these ensure the capacity for good radical stabilization, which is achieved by delocalization of the unpaired electron in the aroxyl radical structure [129].

In general, the B-ring hydroxylation pattern is regarded as being the most significant determinant [130–133], whereas the rest of the molecule seems to become more important only in cases in which only one hydroxyl group in ring B is present or if the hydroxyl group at position 4′ is inactivated by methylation [126]. The A-ring hydroxylation pattern in contrast seems to correlate little with antioxidant activity [124]. A free hydroxyl group in position 3 (ring C) increases the antioxidant activity by the formation of intramolecular hydrogen bonds with the B-ring. This influences the torsion angle of the B-ring with respect to the free molecule and results in a planar molecule, which allows conjugation, electron dislocation and an increased radical stability [124,134]. Flavonols seem to have the highest antioxidant activities and are also easily available commercially, which is well-reflected by the fact that they represent the best studied compounds [27].

Some flavonoids also show prooxidant activities and these also depend on the number of hydroxyl substituents and on the presence of a conjugation system between rings A and B [135]. Pyrogallol structures (3 adjacent hydroxyl groups) in rings A and B seem to be particularly effective in promoting pro-oxidant activity [124]. Thus, antioxidant and pro-oxidant activity of flavonoids are triggered to some extent by the same structural attributes; however, their pro-oxidant activity largely depends on the presence of divalent Cu or Fe ions.

**Figure 15.**
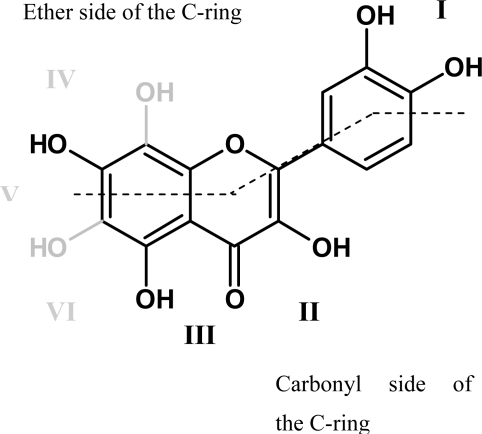
Possible metal binding sites of flavonoids modified from [130].

Flavonoids are well-investigated chelators that frequently bind metal ions in the bidentate mode. Chelation of transition metal ions is an important characteristic contributing to a high antioxidant activity. The most common flavonoids have two to three potential metal-binding sites (I–III in Figure 15), but rare structures with additional hydroxyl groups in the A-ring may have up to six (IV-VI in Figure 15). Keto groups show a lower metal affinity, therefore site I is preferred over II and III. However, site II is the most likely chelation site for Fe ions [136]. Methylated or glucosylated phenol groups are not able to bind metals. Flavonoids with more than one metal-binding site can simultaneously chelate the corresponding number of metal ions, but vicinal sites, e.g., II and III, cannot be used at the same time. Depending on the pH and the concentration of both metal and ligand 1:1, 2:1 and 3:1 complexes can be formed. Thus, oligomeric and polymeric structures can occur in the presence of suitable flavonoid ligands and metal ions, which results in aggregation effects and a decreased ability of to pass through membranes [128]. Complexes of flavonoids with transition metal ions (Fe^2+^, Fe^3+^, and Cu^2+^) have significantly higher radical scavenging properties e.g., superoxide dismutase activity [137,138].

The capacity for metal chelation also results in the inhibition of enzymes with metal ion(s) in the catalytic domain, e.g., the copper-containing metalloenzyme polyphenol oxidase [130]. The highest inhibition (lowest IC_50_ values) is observed with 8-hydroxyluteolin, which can simultaneously interact with metal ions at sites I and IV (Figure 15). In contrast, flavonoids carrying an additional hydroxyl group in position 6 and therefore possessing a comparable dual binding site (I/V and I/VI respectively), show only a low inhibitory activity [130]. Moreover, the hydroxyl group in position 3 drastically reduces the inhibitory activity and therefore flavones are better polyphenol oxidase inhibitors than flavonols. Thus, the inhibitory effect depends on the number of hydroxyl groups on the ether side (see Figure 15) of the flavonoid molecule rather than on the total number of hydroxyl groups.

### 5.3. Relationship between the Structure and Antiinfective Effects

The antiinfective activity of flavonoids has been largely investigated [35]. It seems to be based on a diverse range of effects, and there is no clear and general correlation between the hydroxylation pattern of flavonoids and their antiinfective activity [35]. However, in some cases such a relation was reported. Thus, the antibacterial activity of flavanones against methicillin-resistant *Staphylococcus aureus* and cariogenic *Streptococcus* sp. depends on a *m*-dihydroxy pattern in rings B and A [35,139]. The antimicrobial activity of isoflavonoids and dihydrochalcones was reported to be linked to the number of hydroxyl groups [140,141]. Likewise, monomeric and condensed flavanols with a higher degree of hydroxylation in their B-rings showed increased anthelminitic effects [114,116].

In the pathogen defence of plants, there is a prominent example for flavonoid-type phytoalexins with a specific hydroxylation pattern – particularly in ring C. These flavonoids are characterised by a lack of hydroxyl group in position 3. 3-Deoxyanthocyanidins are formed *de novo* after pathogen attack in a limited number of plants, e.g., sorghum and sugar cane [142–145]. As a rule, the tissues accumulate common 3-hydroxyanthocyanins, which are formed by a light-dependent gene set, whereas 3-deoxyanthocyanins are synthesized only in the case of infection by a separate gene set which is regulated independently of light [143,144,146–149]. The possible contribution of flavan-4-ols, which are the immediate precursors for 3-deoxyanthocyanidin formation, to the observed antimicrobial activity of 3-deoxyanthocyanidin based phytoalexins *in vivo* has not yet been investigated. Strong antimicrobial activity was reported for luteoforol (flavan-4-ol with a 3′,4′-hydroxylation pattern, Figure 9) [150,151]. The lack of comparable data for the corresponding flavan-3,4-diols is mainly based on the instability of these compounds. Compared with catechin, 3-deoxycatechin (luteoliflavan) did not show enhanced antimicrobial activity with respect to *Erwinia amylovora* [152,153].

## 6. Outlook

The hydroxylation pattern of flavonoids contributes significantly to the diversity of natural flavonoid structures. Although flavonoid biosynthesis is well established and the hydroxylation reactions were largely studied, the establishment of rare structures still remains a puzzle. In many cases the number and/or position of hydroxyl groups in the flavonoid structures significantly influence the physiological and biological effects on plants, animals and humans. However, the impact depends on the underlying mode of action, which is in many cases unknown. Furthermore, the availability of systematic studies of the influence of hydroxylation patterns on observed biological effects is limited. It may be assumed that the lack of commercially available reference compounds is one of the reasons for this. The ongoing elucidation of hydroxylation steps in the flavonoid pathway and the identification of corresponding genes may in future allow a biotechnological production of rare flavonoid structures. In addition it is an essential pre-requisite to study the possibilities and limitations of managing phenol contents in crop plants by phytochemical farming and breeding.
